# Operational challenges of the Philippine Antimicrobial Resistance Surveillance Program during the COVID-19 pandemic

**DOI:** 10.5365/wpsar.2022.13.3.917

**Published:** 2022-08-25

**Authors:** Karis Boehme, Sonia B Sia, Ferissa Ablola, June Gayeta, Ma Cecilia Alea

**Affiliations:** a Antimicrobial Resistance Surveillance Reference Laboratory, Research Institute for Tropical Medicine, Department of Health, Manila, Philippines.; * These authors contributed equally.

## Abstract

**Problem:**

Operation of the Philippine Antimicrobial Resistance Surveillance Program (ARSP) has been affected by the coronavirus disease 2019 (COVID-19) pandemic, during which time difficulties in maintaining laboratory functions, staffing levels and participation were reported.

**Context:**

The COVID-19 pandemic has increased pressure on most health systems and programmes in the Philippines, including ARSP. As ARSP is the source of national data on antimicrobial resistance (AMR) trends, there are concerns that the negative effects of the pandemic may have impacted the quality of data produced.

**Action:**

We describe disruptions to laboratory operations, personnel availability and participation in ARSP surveillance, and their impact on reported data for 2020.

**Outcome:**

Surveillance operations were challenged by reallocation of human, infrastructure and financial resources for pandemic response among both the sentinel sites and the coordinating laboratory, the Antimicrobial Resistance Surveillance Reference Laboratory. There was a decrease in the amount of data submitted to the surveillance system, as well as in the number of isolates sent to the reference laboratory for confirmation of bacterial identification and antimicrobial susceptibility testing. Nevertheless, overall performance scores of the sentinel sites for most parameters were comparable to 2019, the year before the pandemic.

**Discussion:**

The impact of operational changes to ARSP due to the pandemic needs to be considered when analysing AMR surveillance data from 2020. Automation of data submission, good working relationships between the coordinating laboratory and sentinel sites, and supply chain system strengthening were identified as key to maintaining AMR surveillance during the COVID-19 pandemic.

## PROBLEM

By the end of 2020, the coronavirus disease 2019 (COVID-19) pandemic had caused around 3.2 million excess deaths worldwide and continues to pose major challenges globally. (*1*) In the Philippines, there have been 474 064 confirmed COVID-19 cases and 9244 deaths (as of the end of 2020), (*2*) raising concerns that the pandemic may accelerate antimicrobial resistance (AMR) in health-care facilities as a consequence of possible antibiotic overuse and misuse. (*3*, *4*) The full effect of the COVID-19 pandemic on AMR will only be observed in the upcoming years through continued AMR surveillance at all levels.

During a pandemic, it is likely that routine surveillance for AMR monitoring, if not prioritized, may contain gaps and missing data. (*5*) This paper describes the challenges posed by the COVID-19 pandemic to the operations of the Antimicrobial Resistance Surveillance Program (ARSP) in the Philippines, from January to December 2020.

## CONTEXT

ARSP is a sentinel, laboratory-based surveillance network comprising 24 sentinel sites located in 16 of the  Philippines’ 17 regions, and a further two sites for gonococcal surveillance. Data from both the regular and gonococcal sentinel sites are presented. The coordinating laboratory – the Antimicrobial Resistance Surveillance Reference Laboratory (ARSRL) – is based at the Research Institute for Tropical Medicine (RITM). Case finding is based on specimens sent to sentinel sites’ clinical laboratories for diagnostic purposes.

RITM has been at the forefront of the Philippine COVID-19 response for both patient care and laboratory testing. From January to June 2020, RITM served as the primary COVID-19 testing centre for the Philippines, responsible for more than 90% of all COVID-19 tests conducted in the country. In March 2020, to accommodate the surge in demand for reverse transcription polymerase chain reaction (RT–PCR) testing for severe acute respiratory syndrome coronavirus 2 (SARS-CoV-2), ARSRL, together with RITM’s other laboratories, was directed to assist the national testing effort by undertaking some of the procedures required, such as sample inactivation and DNA extraction. This directive resulted in the reassignment of ARSRL laboratory staff to the COVID-19 testing team. Facilities, workspaces, equipment and critical resources (reagents, supplies, consumables and personal protective equipment [PPE]) were also repurposed to meet the demand for COVID-19 testing. Directives were also made to postpone scheduled trainings, monitoring visits and face-to-face meetings – activities usually performed by the reference laboratory – until the end of June 2020. Consequently, the number of laboratory technicians who underwent training for AMR bacteriology procedures decreased from 27 to 6.

By July 2020, sufficient additional staff had been recruited to cover the COVID-19 response tasks, allowing existing reference laboratory staff to return to their routine AMR surveillance work. However, until the end of 2020 about 20% of ARSRL laboratory space remained allocated to the COVID-19 response. The annual ARSP Program Implementation Meeting (ARSP PIM) was held virtually via Zoom instead of the usual face-to-face format and the publication of the ARSP 2019 annual surveillance report was delayed by 5 months.

## ACTION

The impacts of the above-mentioned institutional directives on the operations of RITM’s ARSRL were reviewed. In addition, the challenges experienced by the surveillance sentinel sites participating in ARSP during the course of 2020 were summarized.

To assess the impact of the pandemic, ARSP performance indicators (and corresponding targets for participation and number of isolates reported to the surveillance system) for 2020 were compared to those for 2019 (**Table 1**). The set of indicators and targets had been jointly agreed upon by the sentinel sites and ARSRL during previous annual meetings. Information about how the COVID-19 pandemic had affected sentinel site participation in ARSP was gathered from the site status reports that were presented at the ARSP PIM, in particular, the responses to the following questions that sites had been asked to provide: 1) How has the COVID-19 pandemic affected your laboratory operations? and 2) How has the COVID-19 pandemic affected your participation in ARSP?

**Table 1:** Performance scores of the Philippine Antimicrobial Resistance Surveillance Program by sentinel site, 2019 and 2020

Performance indicator	Description	Target (%)	2019 (%)	2020 (%)
On-time submission of regular data	Percentage of data sent on time to ARSRL on a monthly basis	90	68	68
Completeness of demographic data	Percentage of data with the minimum demographic data requirements for reporting	95	94	93
Referral of isolates	Percentage of isolates referred according to the list agreed upon during the ARSP annual meeting (includes pathogens of public health importance based on CLSI and WHO recommendations)	90	48	52
Concordance in identification	Percentage of referred isolates with correct bacterial identification confirmed by the ARSRL (different targets are set for genus and species levels)	-	-	-
4.1 Genus level	Concordance at the genus level	95	98	98
4.2 Species level	Concordance at the species level	87	96	97
Concordance in AST	Percentage of discordant antimicrobial susceptibility results from the sites compared with AST results from ARSRL	-	-	-
5.1 Critical deviations	AST discordance for resistant and susceptible results only	£5	3	3
5.2 Total deviations	AST discordance for resistant, susceptible and intermediate results	£10	7	7
Inclusion of working diagnosis	Percentage of data that includes a working diagnosis (disease indicated by physician’s examination that prompted the request for culture and sensitivity testing)	60	68	66
Completeness of antibiotic panel	Percentage of AMR surveillance data that follows the antibiotic panel agreed upon in the most recent ARSP annual meeting	85	59	61
Encoding of negative results	Percentage of negative culture results encoded in WHONET	85	94	94

AMR: antimicrobial resistance; ARSP: Antimicrobial Resistance Surveillance Program; ARSRL: Antimicrobial Resistance Surveillance Reference Laboratory; AST: antimicrobial susceptibility testing; CLSI: Clinical Laboratory Standards Institute.

## OUTCOME

### Laboratory operations

All 26 sentinel sites reported experiencing operational challenges, which varied in type and extent. Most (18/26, 69.2%) of the sentinel sites cited delays in the usual schedule of delivery of reagents and supplies due to transportation issues. Another commonly reported supply challenge stemmed from the new requirement to use extra layers of PPE, which resulted in episodes of low or depleted supplies of PPE at nearly two thirds (17/26, 65.4%) of sites. In some areas, suspension of courier services had reportedly delayed the referral of bacterial isolates to RITM for confirmatory testing.

Half (13/26, 50%) of all sentinel sites reported a decrease in the number of specimens requiring routine culture and sensitivity testing. Four reported periodic closure of their outpatient departments. Sites also reported a low influx of patients, and the two nongovernment sentinel sites experienced a reduction in revenue for the laboratory.

### Laboratory personnel

The majority (14/26, 53.9%) of sentinel sites reported being designated as COVID-19 referral hospitals during the pandemic and having to establish or expand their molecular biology facilities. Even though this provided opportunities for acquisition of new equipment and increasing laboratory staff capacity for molecular detection of pathogens, it also meant temporary reallocation of space and human resources for RT–PCR testing for COVID-19.

The majority of sentinel sites also reported encountering various challenges relating to laboratory personnel. Seventeen sites (65.4%) experienced a reduction in the number of active-duty staff over several periods due to reassignment to other hospital units for COVID-19 sample processing and testing. Furthermore, around one third (9/26, 34.6%) reported that on several occasions staff work schedules had to be reduced to a skeletal workforce to maintain physical distancing in the laboratory. Staff in high-risk groups were ordered to work from home, further decreasing the number of staff available to work in bacteriology laboratories. Eleven sentinel sites (42.3%) reported that they were provided with additional manpower to help overcome staffing challenges.

The health status of frontline workers at ARSP sites was also affected, with some infected with COVID-19 and some experiencing anxiety because of the requirement to be at work. Laboratory personnel from four sites in the National Capital Region (NCR) reported experiencing difficulties in getting to work due to travel restrictions and suspension of transport services.

### Participation in ARSP

Delays in transporting isolates to ARSRL for confirmatory testing, typically because of the lack of courier services, was a common challenge among sentinel sites (9/26, 34.6%). Moreover, the decrease in the number of samples requiring testing at sentinel sites also reduced the demand for confirmatory testing of isolates.

Although automated transmission of data from the sentinel sites to ARSRL had already been established before the pandemic, many sentinel sites still reported experiencing delays in encoding identification and susceptibility data to WHONET due to decreased staff numbers in their bacteriology sections.

### ARSP performance indicators

Of the 10 reported performance indicators, five were unchanged between 2019 and 2020 (**Table 1**). These were: on-time submission of regular data (1), concordance in identification (genus level) (4.1), concordance in antimicrobial susceptibility testing (AST) (critical deviations) (5.1), concordance in AST (total deviations) (5.2), and encoding of negative results (8). There were decreases for completeness of demographic data (2) and inclusion of working diagnosis (6), but increases for referral of isolates (3), concordance in identification (species level) (4.2), and completeness of antibiotic panel (7).

### Comparison of ARSP data submission: 2019 versus 2020

Across all sentinel sites, the number of AST data submissions dropped by 38.7%, from a total of 100 334 in 2019 to 61 527 in 2020 (**Table 2**). All sentinel sites in the NCR recorded substantial decreases in reported data, with two sentinel sites unable to submit any AST data at all in 2020. Relative to the NCR, sites in Visayas had smaller decreases in test data submissions, with one site reporting a 5% increase. In Mindanao, a decrease was observed in four out of the six sites (**Table 2**).

**Table 2:** Number of isolates with antimicrobial susceptibility testing data submitted to the Philippine ARSP by sentinel sites in 2019 and 2020, and percentage change from 2019

Region/sentinel site	2019	2020	% change
Luzon – National Capital Region			
Site 1	548	0	–100.00
Site 2	4358	0	–100.00
Site 3	2371	1019	–57.02
Site 4	13 895	6818	–50.93
Site 5	507	255	–49.70
Site 6	2722	1419	–47.87
Site 7	4433	2713	–38.80
Site 8	2375	2027	–14.65
Luzon – outside National Capital Region			
Site 9	90	13	–85.56
Site 10	3633	1569	–56.81
Site 11	2521	1176	–53.35
Site 12	5234	2968	–43.29
Site 13	4824	3248	–32.67
Site 14	5668	3782	–33.27
Site 15	4462	3581	–19.74
Visayas			
Site 16	2539	1425	–43.88
Site 17	3957	2624	–33.69
Site 18	10 286	6886	–33.05
Site 19^a^	352	289	–17.90
Site 20	3874	4056	+4.70
Mindanao			
Site 21	12 177	7412	–39.13
Site 22	3181	2076	–34.74
Site 23	1205	1115	–8.07
Site 24	3409	3735	+9.56
Site 25	1644	1192	–27.49
Site 26^a^	69	129	+86.96
**TOTAL**	**100 334**	**61 527**	**–38.68**

^a^ Gonococcal surveillance sites.

### Lessons learnt

Sentinel sites that submitted the same or increased volumes of AMR surveillance data in 2020 were asked to explain how they were able to maintain their 2019 levels of performance despite the pandemic. At Site 26, a gonococcal surveillance site, scheduled testing for sex workers continued despite the pandemic, resulting in an increase in submissions, a commendable achievement and one that highlights the importance of retaining AMR surveillance for gonococcal infections. Site 20s increase in submitted data may have been due to the increase in the number of admissions due to COVID-19 infections, which prompted an increase in requests for AST, especially for those admitted for respiratory symptoms. Site 24 reported that they were able to mitigate challenges related to procurement of laboratory reagents and supplies by strengthening communications and coordination with suppliers regarding possible delays, changes and expiration dates of goods for delivery.

Practices that proved useful in overcoming challenges caused by the COVID-19 pandemic included prompt preparation of a procurement plan for reagents and supplies for COVID-19 testing, which helped minimize the risk of depletion of supplies; establishment of a process for sharing laboratory supplies among the reference laboratory and sentinel sites; and use of logistics created for the COVID-19 pandemic to support AMR surveillance (i.e. some sentinel sites were able to send both isolates for AST and samples for COVID-19 testing to RITM simultaneously).

## Discussion

The challenges to ARSP laboratory operations, including staffing, experienced during the COVID-19 pandemic did not appear to reduce overall participation of the sentinel sites in ARSP. This could be due, at least in part, to the fact that all sentinel sites, having been involved in ARSP for more than 5 years, have surveillance activities ingrained in their operations such that the disruptions of the COVID-19 pandemic did not result in lessened participation in surveillance activities. However, we recognize the limitation that the activity reports provided by each site may not have included other relevant aspects of laboratory operations. Estimated changes in the performance indicators reported here cannot therefore be conclusively attributed to the actions described by the sentinel sites in their activity reports.

The good working relationships between RITM and sentinel site personnel, formed through partnerships that have been in place since 1988, may have been a contributory factor in maintaining high levels of participation in ARSP. The practice of recognizing and incentivizing sites with top participation scores, which was established in 2012, may have also encouraged continued participation in ARSP during the COVID-19 pandemic.

The process of automated data transfer, whereby WHONET encoded data from the sentinel sites are automatically transmitted to the ARSRL server, facilitated the data submission from sentinel sites throughout 2020. ARSRL, as the coordinating laboratory, adapted to the challenges caused by the pandemic by adjusting staff schedules and activities, holding the annual PIM online and delaying the publication of the annual surveillance report.

There was, however, a reduction in the amount of surveillance data submitted in 2020, a factor that must be considered when interpreting the overall AMR rates reported by ARSP for 2020. It is possible that the decrease in submitted data was due to the shift towards remote outpatient consultations (to lessen the risk of infection at the sentinel sites), a trend which could introduce bias in patient and testing denominators for the 2020 ARSP surveillance data, and which should be considered in the analysis of AMR data. (*6*, *7*)

The actions implemented by the participating sentinel sites alleviated much of the negative impact of the pandemic on laboratory operations and logistics. Performance indicators revealed that despite the ongoing health crisis, sentinel sites were able to perform their tasks as the primary contributors to national AMR data collection. It is imperative for ARSRL to disseminate information and encourage other facilities to adopt the good practices observed at these sites. The success and continuity of ARSP is contingent upon the collaborative efforts of the reference laboratory and the sentinel sites. The COVID-19 pandemic has the potential to exacerbate the AMR situation in the Philippines and put stewardship efforts at risk. It is imperative therefore that efforts against the development of AMR should not cease.

Further studies should be conducted to provide more information on the impact of the COVID-19 pandemic on AMR emergence and spread. Moreover, the expanded molecular biology facilities established to meet the needs of the COVID-19 response should be used to enhance national AMR surveillance through genomic epidemiology.
